# Individual Circadian Preference, Eating Disorders and Obesity in Children and Adolescents: A Dangerous Liaison? A Systematic Review and a Meta-Analysis

**DOI:** 10.3390/children9020167

**Published:** 2022-01-28

**Authors:** Francisco José Rodríguez-Cortés, Ignacio Morales-Cané, Pedro Manuel Rodríguez-Muñoz, Rosaria Cappadona, Alfredo De Giorgi, Roberto Manfredini, María Aurora Rodríguez-Borrego, Fabio Fabbian, Pablo Jesús López-Soto

**Affiliations:** 1 Department of Nursing, Instituto Maimónides de Investigación Biomédica de Córdoba, 14005 Cordoba, Spain; francisco.rodriguez@imibic.org (F.J.R.-C.); n82mocai@uco.es (I.M.-C.); pedromrodriguez@usal.es (P.M.R.-M.); en1robom@uco.es (M.A.R.-B.); pablo.lopez@imibic.org (P.J.L.-S.); 2 Department of Nursing, Pharmacology and Physiotherapy, Universidad de Córdoba, 14004 Cordoba, Spain; 3 Department of Nursing, Hospital Universitario Reina Sofía de Córdoba, 14004 Cordoba, Spain; 4 Department of Nursing and Physiotherapy, Universidad de Salamanca, 37007 Salamanca, Spain; 5 Department of Medical Science, University of Ferrara, 44121 Ferrara, Italy; rosaria.cappadona@unife.it; 6 Clinica Medica Unit, Azienda Ospedaliero-Universitaria ‘S.Anna’, 44121 Ferrara, Italy; degiorgialfredo@libero.it; 7 University Center for Studies on Gender Medicine, University of Ferrara, 44121 Ferrara, Italy

**Keywords:** feeding and eating disorders, obesity, chronobiology, phenomena, chronotype, circadian rhythms, adolescents, children, sex, gender

## Abstract

Background: Obesity and other eating disorders are an actual public health problem, especially in childhood and adolescents, and could be also related with chronotype. The aim of this systematic review was to determine the relationship between eating disorders, obesity and the different chronotypes in children and adolescents. Methods: A systematic review of observational studies evaluating young populations dealing with and evaluating chronotype was conducted. Electronic searches were performed in six international databases. A qualitative thematic-categorical analysis was carried out and a random-effects model was used for the quantitative analysis (meta-analysis). Results: Fifteen studies were included, but quantitative analysis was only carried out in three of them. Children and adolescents with an evening chronotype had higher body mass index, consumed more junk food or were more predisposed to suffer from food addiction and night eating syndrome. Conclusions: Children and adolescents with evening chronotype had higher tendency to incorrect eating behaviors and were suffering from overweight/obesity. Environment but also lifestyle factors should be considered in the association between chronotype and eating disorders and obesity.

## 1. Introduction

The behaviors and biological rhythms of each individual are determined by circadian variations associated with the light-dark cycle over a period of about 24 h per day [1,2]. Possible individual differences in circadian attitudes are called ‘chronotype’. According to Horne and Ostberg, five categories are differentiated: definitely Evening type (E-type), moderately Evening type, neither type or Intermediate (I-type), moderately Morning type (M-type), and definitely Morning type [3,4]. The morning chronotype shows the preference of individuals to wake up early and perform activities early. In contrast, the evening chronotype is found in individuals who wake up late and perform activities in the afternoon. Finally, the intermediate chronotype is found between the two chronotypes [3,5].

At least for people living in the temperate regions, data seem to indicate that chronotype respects a Gaussian distribution, with 10% M-type, 10% E-type, 80% I-type [6]. However, the chronotype is not the same throughout an individual’s life. Thus, under normal conditions, as the individual advances in age, a shift from the evening chronotype to the morning chronotype is observed [7].

The relationship between the presence of one chronotype or another and different health problems is currently being studied in greater depth. Of the chronotypes identified, individuals with an E-type chronotype have shown a greater tendency to present health problems, as well as a higher mortality rate compared to individuals with a M-type chronotype [8,9] This is the case for mental disorders, such as anxiety and depression, as well as for endocrine–metabolic disorders such as obesity or diabetes, cardiovascular diseases, sleep disorders, and eating disorders, such as nocturnal eating syndrome (NES) [2,10]. With particular reference to NES, a syndrome recently included in the Diagnostic and Statistical Manual of Mental Disorders 5, the relevant role of chronotype in its occurrence has been observed [11,12].

The determination that different clinical conditions could be associated with E-type suggested that chronotype should be considered as an important factor for improving lifestyle and prevention as well [5,13]. However, most of the evidence has focused on the adult population and to a lesser extent on adolescents and children. 

In developed countries, childhood obesity and other eating disorders have increased considerably and have become a public health problem. Obesity has a multifactorial etiology and is determined by lifestyles adopted during childhood and adolescence [14]. The present systematic review seeks to assess the current scientific evidence on the relationship between eating disorders, obesity, and different chronotypes in studies investigating children and adolescents.

## 2. Materials and Methods

### 2.1. Design, Search Methods and Inclusion/Exclusion Criteria

A systematic review of primary studies indexed in different databases was conducted to ascertain the available scientific evidence. Thus, a qualitative and quantitative synthesis of the findings was made and the quality of the evidence of the included studies was assessed. The Preferred Reporting Items for Systematic Reviews and Meta-Analysis (PRISMA) checklist [15] was used to write the review, following these criteria: (i) formulation of the research question, using the PICO (patient, intervention, comparison and outcome) format; (ii) definition of the search strategy; (iii) data extraction; and (iv) analysis and interpretation of the data obtained and assessment of the quality of the selected articles. The Cochrane Handbook (version 6.1) [16] was used as a guide in the review process. The protocol of this review was registered in PROSPERO (CRD42021285296) [17].

The databases used for the search were: Medline (through PubMed), EMBASE, Web of Science, EBSCO, and Cochrane Library. A systematic search strategy was developed, searching for keywords related to the categories “eating disorders”, “obesity”, and “Chronotype”; to combine them, the Boolean operators “AND” and “OR” were used. The search strategy was adapted to each of the databases (Supplement Table S1).

The following inclusion criteria were considered: (a) observational studies conducted worldwide; (b) papers published between 1 January 2011 and 30 June 2021; (c) mean age of the populations between 3 and 21 years; (d) determination of the chronotype in the presence of obesity and/or eating disorders. Articles that did not meet any of the above inclusion criteria were excluded. The search was not limited by language. The search was limited to the last 10 years with the intention of finding the most recent evidence, and 30 June 2021 was the last definitive access.

### 2.2. Study Selection, Strategy for Data Synthesis and Quality Assessment

Two researchers (FJRC and IMC) independently assessed all references identified in the search. First, we screened according to the title and abstract. Subsequently, articles that met the inclusion criteria in the first phase were read in full text to determine their final inclusion. In cases where there was disagreement between the two researchers on the inclusion of a manuscript, a third researcher (PJLS) was consulted.

A qualitative and quantitative synthesis of the data was conducted regarding the findings of the included articles, depending on the nature of the data retrieved. A narrative data synthesis of the variables of the included studies was performed: socio-demographic characteristics of participants, chronotype (E-type vs. M-type) and outcome results (eating disorders, obesity, and clinical and socio-demographic factors associated) (Table 1).

Otherwise, when included studies were sufficiently homogenous in terms of outcomes and the measurement of outcomes (exclusively for obesity-related articles), the results were presented as a quantitative synthesis of aggregate participants data and statistically studied in a random-effects model of meta-analysis. Standardized mean differences, 95% confidence intervals, and two-sided *p*-values were used. Heterogeneity between the studies in effect measures was assessed using both the I^2^ test and the I^2^ statistic. A I^2^ value greater than 50% was indicative of substantial heterogeneity. Analyses were performed with RevMan software (version 5.3.5).

Study quality was assessed using the Quality Assessment for Observational Cross-Sectional Studies and Quality Assessment of Case-Control Studies questionnaires of the National Heart, Lung, and Blood Institute, last updated in 2021 [31]. The quality was related to the number of items found in each study, being good if 0 or 1 item was not met and being fair if more than 3 items were not met. 

## 3. Results

**Figure 1:**
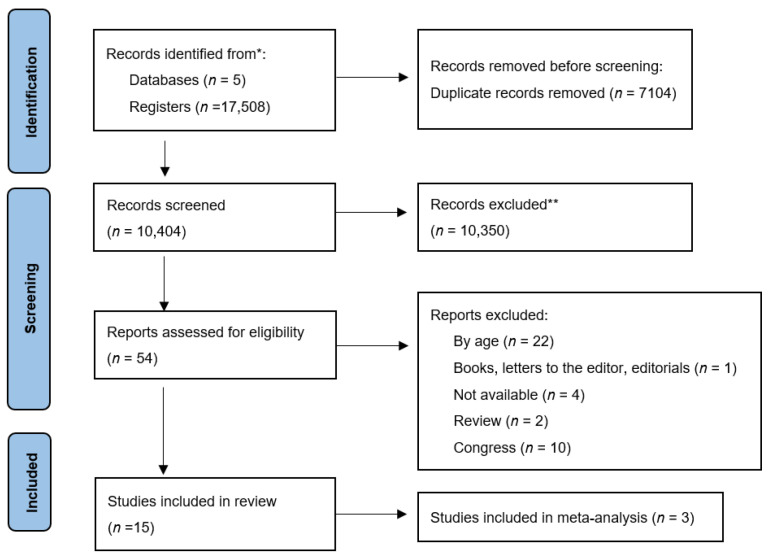
PRISMA study selection flowchart. * Sum of the manuscripts found in the six databases; ** Not meeting the inclusion criteria after reading the title and abstract.

Figure 1 illustrates the study selection process. The search yielded a total of 17,508 records, of which 7104 were duplicates (same manuscript found in different databases), resulting in a total of 10,404 unique records. After review, 54 were included for full text reading. Finally, 15 met the criteria for inclusion.

### 3.1. General Characteristics of the Studies

Twelve of the included studies had a cross-sectional design [10,12,18,20,21,22,23,24,26,27,29,30], one was a retrospective cohort [28] and two were case-control [19,25]. Six out of the fifteen studies selected were conducted in Turkey and two in Spain. The remaining seven studies were conducted in Italy, Hong Kong, Lebanon, France, United Kingdom, China, and Germany. The mean age of the study population ranged from 21 [22] to 9 years [28] (Table 1). With regard to the quality of evidence, 6 out of the 15 selected studies were considered as ‘good’, [10,20,22,24,27,28], while the remaining 9 were rated ‘fair’ [12,18,19,21,25,26,29,30] (Tables S2–S4).

#### 3.1.1. Chronotype

The Horne–Ostberg Morning–Evening Questionnaire (MEQ) to determine circadian preference was used by 8 of the 12 cross-sectional articles [10,12,18,21,23,24,26,29], while a different cross-sectional study [22] shortened the Spanish version of MEQ. Two cross-sectional studies [20,30] used the Children’s Chronotype Questionnaire (CCTQ) and the Munich ChronoType Questionnaire (MCTQ) was evaluated in a cross-sectional study [27]. E-type chronotype was associated with higher evening eating scores than M-type and I-type chronotypes as shown by the NEQ questionnaire (*p* < 0.01) [12,29]. E-type was also associated with higher carbohydrate and fat intake (*p* = 0.021, *p* = 0.033, respectively) [18], and increased likelihood of obesity (OR 5.66, Cl 1.91–16.81) [20] especially in females (*p* = 0.05) [21]. The studies by Saidi et al. [23] and Roßbach et al. [27] did not find a direct relationship between chronotype and higher intake of carbohydrates or any macronutrients. However, in the study by Arora et al. [24], a significant association with unhealthy snacking consumption was observed (*p* = 0.012). In addition, the E-type chronotype was associated with low adherence to the Mediterranean diet (*p* < 0.01) and skipping breakfast (*p* < 0.01) [22]. On the other hand, the study by Najem et al. [10] did not find a direct relationship between chronotype and food addiction (FA), but E-type individuals were found to have higher stress levels (*p* = 0.010) [10]. 

Finally, the studies by Yu et al. [28] and Arora et al. [24] showed that E-type boys and girls were less likely to consume fruits and vegetables and more likely to skip breakfast or have a light breakfast (*p* < 0.05). 

#### 3.1.2. Association with Sex

Thirteen studies analyzed samples with individuals of both sexes [10,12,19,20,21,22,24,25,26,27,28,29,30], however, Bodur et al. [18] and Saidi et al. [23] included only females. Only three out of the fifteen articles [20,22,28] conducted sex-specific analyses, and none of them obtained significant results.

#### 3.1.3. Eating Disorders

Regarding eating disorders, five of the seven studies presented data associated with body mass index (BMI) > 25 kg/m^2^ [10,18,20,21,22,25,26,30]. BMI was related to the Yale Food Addiction Scale (YFAS) (*p* = 0.02) [10]. Additionally, Ağagündüz et al. [30] showed that E-type adolescents had lower energy expenditure (*p* < 0.05).

On the other hand, individuals whose parents practiced physical exercise had a lower level of low adherence to the Mediterranean diet (*p* = 0.02) and higher tendency towards erotophilia (positive attitude towards sexual stimulus, with more favorable emotions and evaluations leading to a greater search for sexual stimuli) [22]. Eveningness was associated with lower intake of fruits, vegetables, pulses, cereals, and olive oil, and higher breakfast skipping [22]. In the same lane, Yu et al. [28] reported that boys were prone to skipping breakfast, while girls consumed fast food more frequently (*p* < 0.001).

Two studies [12,29] focused on Night Eating Syndrome (NES) through the Night Eating Questionnaire (NEQ). NEQ scores were reported to have a direct effect on EAT (Eating Attitude Test) scores (β = 0.21; *p* = 0.028) [29] and were associated with Beck Depression Inventory (BDI) scores (r = 0.275; *p* = 0.001) [12]. Concretely, authors concluded that NES could misalign the food intake and then disrupt the circadian rhythms of sleep phase, generating insomnia [12]. However, Esin et al. [19] found that sleep duration and number of psychiatric disorders stood out as the main risk factors, independently of the chronotype. 

In the present review, no articles were found that addressed the chronotype of children and/or adolescents with typical eating disorders, i.e., anorexia nervosa, bulimia nervosa, or binge eating disorder.

#### 3.1.4. Quantitative Analysis: Overweight and Chronotype

**Figure 2:**
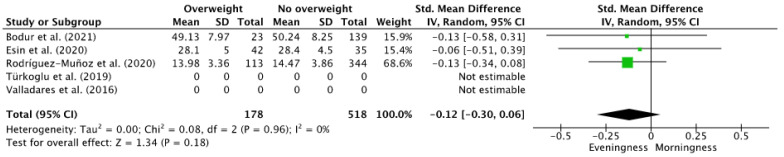
Forest plot of the relationship between chronotype and overweight/obesity.

A quantitative analysis was carried out with three of the eight studies [18,19,22] that addressed the relationship of chronotype with overweight. Individually, differences in chronotype scores were observed between adolescents with BMI > 25 and those with an index below 25. Overall, there was a trend, although not significant (*p* = 0.18) between overweight and E-type chronotype [Standard Mean Difference (95% Confidence Interval) −0.12 (−0.30, 0.06)] (Figure 2). Although different questionnaires were used for chronotype determination, no heterogeneity was observed (I^2^ = 0%).

## 4. Discussion

The present study shows that eating disorders and obesity could be associated with E-type chronotype in children and adolescents, with no definite sex or gender differences. Specifically, E-Type children and adolescents have higher BMI, consume more junk food, or are more predisposed to suffer from food addiction and night eating syndrome. E-type children and adolescents have a higher intake of carbohydrates and fat, lower adherence to the Mediterranean diet, lower consumption of fruit and vegetables, higher likelihood of skipping breakfast, and lower energy expenditure [10,12,18,19,20,21,22,23,24,25,26,27,28,29,30].

The individual’s circadian system adapts physiological processes and behavior to the existing demands of the light-dark cycle. The interaction between the external environment and an individual’s circadian phase establishes the chronotype [32]. In current society, artificial lightning has influenced the distribution of chronotype in the population, specifically in adolescents. In fact, it has been shown that adolescents living in urban areas and exposed to artificial lighting at night tend to have an evening chronotype compared to those living in rural areas [33]. Moreover, a cross-sectional telephone study has been conducted on a representative sample of the general adult population of the United States (>19,000 individuals), combined with Outdoor Nighttime Light (ONL) measurements obtained from the Defense Meteorological Satellite Program [34]. The results showed that living in areas with greater ONL was associated with delayed bedtime and wake up time, shorter sleep duration, and increased daytime sleepiness. On one hand, it is already known that the decline of the total amount of sleep plays a critical role in the increase of obesity, due to a series of mechanisms [35]. On the other hand, the misalignment of sleep time of the evening chronotype is associated with metabolic risk factors [36].

Therefore, environmental but also lifestyle factors can explain partially the association between chronotype and eating disorders and obesity [37]. In line with the results of the present systematic review, several manuscripts carried out in the adult population have associated an evening chronotype with suboptimal eating behavior and physical activity habits. People with evening preferences perform better in the afternoon or evening. This circumstance, together with the schedules “imposed” by society, leads to less adherence to healthy lifestyle habits (e.g., less physical activity because sport practice areas are less crowded or sports centers are not adapted to evening hours; unlike other types of shops, junk food establishments have evening opening hours) [27]. Indeed, E-type has been associated with a high impact in physical and mental health, resulting in lower school achievements [8]. 

The findings of the present review show the heterogeneity of factors associated with the development of eating disorders. Several manuscripts use mediation analyses to find out the direct and indirect effects on the dependent variables [10,26,29]. The use of different questionnaires to detect the chronotype of the participants is also striking. These data make it difficult to develop a quantitative analysis, which is limited to the relationship between chronotype and BMI. In addition, it should be noted that some manuscripts have used BMI z-score or percentile [24,25]. In fact, evidence shows that when using BMI or BMIz alone to define overweight or obesity, there exists an increased risk for misclassification [38].

Childhood obesity is a predictor of adult obesity. In the long term, it is associated with multiple chronic diseases such as cardiovascular disease, cancer, diabetes mellitus, gallbladder disease, as well as other endocrine and metabolic disorders [39].

The high levels of childhood obesity worldwide point to the urgent need to address childhood obesity through a holistic intervention. Interventions need to be aimed at children/adolescents and parents as well as education and health professionals who can positively influence early education. The data on childhood and youth obesity suggest that the most important time for intervention is during school time, because this is where students spend most of their day, and it is during school age that they establish health and lifestyle behaviors that may be difficult to change in the future. Specifically, the need to develop interventions considering chronotype in diet, sleep–wake pattern, or screen time could be important [28,40].

As indicated in the selected studies, dietary habits appear to be strictly connected to chronotype and are progressively consolidated from childhood to adulthood. Strategies to counteract circadian mismatch (social jetlag, meal timing, sleep time) or unhealthy diets (sugary beverage intake, food addiction) are required. In a recent systematic review [41], multi-level interventions reported effectiveness in reducing breakfast skipping prevalence. Not only meal timing is associated with chronotype and obesity, but also higher intake of carbohydrate and fat is described in E-type adolescents [18]. In the same line, Xiao et al. [42] demonstrated that the timing of carbohydrate and protein was related with obesity. Last but not least, chronotype can be influenced by social, cultural, and economic factors [43]. The Mediterranean diet, characterized by high intake of fruit, vegetables, legumes, cereals, and using olive oil as the main lipid, is considered a healthy diet [44]. Several studies report that M-type adolescents have a higher adherence to the Mediterranean diet [22,45] and consume low industrially processed foods.

### Limitations

Although a comprehensive search strategy has been employed, there is a possibility that studies of interest may have been missed as only peer-reviewed studies were included and gray literature, dissertations, and conference abstracts were not considered. A possible further limitation could be the duration of our searching strategy. However, we decided to limit our literature search to the last decade, since we intended to provide data on the most recent evidence. Thus, the last access to the databases was on 30 June 2021.

## 5. Conclusions

The findings obtained in the present review indicate that children and adolescents with late chronotype have a higher tendency to suboptimal eating behaviors. Moreover, the qualitative and quantitative analysis shows a greater tendency for the late chronotype to be more overweight and obese.

Environment but also lifestyle factors can explain partially the association between chronotype and eating disorders and obesity. Therefore, strategies or holistic interventions aimed at children/adolescents and parents as well as education of health professionals are required. Concretely, individual interventions considering social, economic, and cultural factors as well as chronotype are required to recognize the relationship between circadian mismatch and unhealthy diets.

## Figures and Tables

**Table 1:** Main characteristics of the selected studies.

Authors, Year	Design (Country)	Disorder (*n*)	Control (*n*)	Gender M/F	Age (SD) [Disorder/Control]	Chronotype (SD) [Disorder/Control]	Tool	Main Results
Bodur et al. (2021) [18]	Cross sectional study (Turkey)	BMI > 25 23	139	0/23 0/139	[20.13 (1.55) years 20.39 (1.53) years]	[49,13 (7.97) 50,24 (8.25)]	MEQ	E-type individuals have a lower intake of fruit and a higher intake of refined grains. Healthy individuals may be vulnerable to chronic diseases. In the long-term, chronotype effect in dietary (high energy, carbohydrate and fat intake of E-type individuals)
Esin et al. (2020) [19]	Case-control study (Turkey)	BMI > 25 42	35	20/22 20/15	[11.5 (2.9) years 10.4 (2.9) years]	[28.1 (5) 28.4 (4.5)]	CCTQ	No risk factors (chronotype or having at least one psychiatric disorder) were statistically associated to become obese.
Türkoğlu et al. (2019) [20]	Cross sectional study (Turkey)	BMI > 25 22	56	22/0 56/0	10.02 (2.24) years	NR	CCTQ	E-type was directly related to obesity and M-type related to normal weight in children and adolescents with attention deficit hyperactivity disorder
Valladares et al. (2016) [21]	Cross sectional study (Spain)	BMI > 25	65	30/35	20 years	[48.5 49.1]	MEQ	E-type is strongly associated with altered body composition measures. E-type may be a risk factor for the development of metabolic diseases.
Rodriguez-Munoz. et al. (2020) [22]	Cross sectional study (Spain)	BMI > 25 113	344	152/305	22 (4) years	[13.98 (3.36) 14.47 (3.86)]	rMEQ	Having an E-type during university was associated with poor adherence to the Mediterranean diet. Chronotype is an important variable that interferes with diet and sexual opinion.
Saidi et al. (2020) [23]	Cross sectional study (France)	BMI > 25 and E-type 16	NA	0/16	13.18 (0.98) years	NA	MEQ	E-type adolescent women with obesity, after practicing intense exercise, show a decrease in the intake of foods with high energy content.
Arora et al. (2015) [24]	Cross sectional study (United Kingdon)	BMI z-score (NR)	NA	212/299	12.0 (0.7) years	NR	MEQ	E-type are associated with a high BMI and poorer eating behaviors. There is also a negative relationship between sleep duration and BMI
Karadag et al. (2021) [25]	Case-control study (Turkey)	BMI ≥ 95th percentile 79	82	41/38 45/37	[12.1 (2.3) years 12.4 (2.2) years]	31.9 (6.3) 27.2 (5.7)	CCTQ	Obese children and adolescents had greater evening preference, longer sleep debt duration, SJL duration and SJLsc duration, higher MEQ scores, and shorter mean sleep duration
Li et al. (2018) [26]	Cross sectional study (China)	Sugary beverage intake (NR)	NA	271/517	19.8 (1.1) years	M-type: 21.8% I-type: 62.8% E-type: 15.4%	MEQ	Chronotype and sleep duration were associated with BMI, and this relationship was mediated by sugary beverage intake.
Roßbach et al. (2018) [27]	Cross sectional study (Germany)	BMI (According to IOTF)	NA	184/162	12.2 (1.4) years	Median (Q1; Q3)	MCTQ	E-Type adolescents are more likely to perform regular breakfast skipping and higher evening energy intake.
Yu et al. (2020) [28]	Retrospec. cohort study (Hong Kong)	Fast Food intake 398	98	261/235	9.25 (1.58) years	Boys: - M-type: 74.2% - E-type: 91.2% Girls - M-type: 43.5% - E-type: 86.2%	CCTQ	E-type boys were more likely to eat fast food than M-type boys [OR = 3.62 (1.07–12.26), *p* = 0.03]. When adjusted for the screen time of the children no statistically significant differences were found [aOR = 3.18, (0.91–11.16), *p* = 0.07]
Najem et al. (2020) [10]	Cross sectional study (Lebanon)	Food addiction 65	579	190/453	20.22 (1.8) years	47.89 (8.03) - M-type: 8.7% - E-type: 20.5%	MEQ	Poor sleep quality, high stress and an E-type could lead to the development of food addiction, as their effect can be cumulative.
Kandeger et al. (2018) [29]	Cross sectional study. (Turkey)	NES 20	363	9/11 153/230	[20.55 (2.46) years 21.12 (2.31) years]	[45.25 (10.67) 51.24 (8.56)]	MEQ	Presence of NES affects chronotype differences and insomnia severity. NES might represent a misalignment of food intake and might shift the chronotype to the late sleep phase as a peripheral oscillator in humans.
Riccobono et al. (2019) [12]	Cross sectional study. (Italy)	NES 12	289	114/175	[17.25 (1.54) years 17.65 (1.29) years]	[42.92 (11.1) 47.56 (8.39)]	MEQ	A high prevalence of NES was observed in subjects with an E-type and depression.
Ağagündüz et al. (2020) [30]	Cross sectional study (Turkey)	REE 103	NA	57/46	10.6 (2.19) years	31.5 (7.39)	CCTQ	Compared with M-type and I-type, participants with E-type had lower energy expenditure for physical activity, lower REE and total energy expenditure.

NR—Not reported; BMI—Body Mass Index; MEQ—Morningness–Eveningness Questionnaire; rMEQ—reduced Morningness–Eveningness Questionnaire; CCTQ—Children’s Chronotype Questionnaire; NA—Not applicable; SJL—Social JetLag; SJLsc—sleep-debt corrected SJL; MCTQ—Munich ChronoType Questionnaire; REE—Resting Energy Expenditure; NES—Night Eating Syndrome.

## Data Availability

The data presented in this study are available on request from the corresponding author.

## Supplementary Materials

The following supporting information can be downloaded at: https://www.mdpi.com/article/10.3390/children9020167/s1, Table S1: Search strategy; Table S2: Quality Assessment for Observational Cross-sectional Studies (National Heart, Lung, and Blood Institute, 2021); Table S3: Quality Assessment for Observational Cross-sectional Studies (National Heart, Lung, and Blood Institute, 2021); Table S4: Quality Assessment of Case-Control Studies (National Heart, Lung, and Blood Institute, 2021).
