# Supplementary material for: Individual Circadian Preference, Eating Disorders and Obesity in Children and Adolescents: A Dangerous Liaison? A Systematic Review and a Meta-Analysis
Source: Children (Basel). 2022 Jan 28;9(2):167. doi: 10.3390/children9020167 (PMC8870066; doi:10.3390/children9020167)
Supplement: Supplementary file 1 [file children-09-00167-s001.zip › children-1559485-supplementary.pdf]

Table S1. Search strategy

Database	Search strategy
Cochrane Library	(((“Feeding and Eating Disorders”):ti,ab,kw OR (“Eating and Feeding Disorders”):ti,ab,kw OR (“Feeding Disorders”):ti,ab,kw OR Disorder, Feeding”):ti,ab,kw OR (“Disorders, Feeding”):ti,ab,kw OR (“Feeding Disorder”):ti,ab,kw OR (“Eating Disorders”):ti,ab,kw OR (“Disorder, Eating”):ti,ab,kw OR (“Disorders, Eating”):ti,ab,kw OR (“Eating Disorder”):ti,ab,kw OR Appetite Disorders”):ti,ab,kw OR (“Appetite Disorder”):ti,ab,kw OR (“Binge-Eating Disorder”):ti,ab,kw OR (“Binge Eating Disorder”):ti,ab,kw OR (“Binge-Eating Disorders”):ti,ab,kw OR Disorder, Binge-Eating”):ti,ab,kw OR (“Disorders, Binge-Eating”):ti,ab,kw OR Night Eating Syndrome”):ti,ab,kw OR (“Eating Syndrome, Night”):ti,ab,kw OR (“Eating Syndromes, Night”):ti,ab,kw OR Night Eating Syndromes”):ti,ab,kw OR Bulimia”):ti,ab,kw OR (“Bulimias”):ti,ab,kw OR Binge Eating”):ti,ab,kw OR (“Eating, Binge”):ti,ab,kw OR (“Food Addiction”):ti,ab,kw OR (“Food Addictions”):ti,ab,kw OR (“Compulsive Eating”):ti,ab,kw OR (“Eating, Compulsive”):ti,ab,kw OR (“Diet”):ti,ab,kw OR (“Diets”):ti,ab,kw OR (“Digestive System”):ti,ab,kw OR (“Alimentary System”):ti,ab,kw OR (“Dyssomnias”):ti,ab,kw OR (“Dyssomnia”):ti,ab,kw OR (“Sleep Disorders, Extrinsic”):ti,ab,kw OR (“Extrinsic Sleep Disorder”):ti,ab,kw OR (“Extrinsic Sleep Disorders”):ti,ab,kw OR (“Sleep Disorder, Extrinsic”):ti,ab,kw OR (“Limit-Setting Sleep Disorder”):ti,ab,kw OR (“Limit Setting Sleep Disorder”):ti,ab,kw OR (“Limit-Setting Sleep Disorders”):ti,ab,kw OR (“Sleep Disorders, Limit-Setting”):ti,ab,kw OR (“Sleep Disorder, Limit-Setting”):ti,ab,kw OR (“Sleep Disorder, Limit Setting”):ti,ab,kw OR (“Nocturnal Eating-Drinking Syndrome”):ti,ab,kw OR (“Eating-Drinking Syndrome, Nocturnal”):ti,ab,kw OR (“Eating-Drinking Syndromes, Nocturnal”):ti,ab,kw OR (“Nocturnal Eating Drinking Syndrome”):ti,ab,kw OR (“Nocturnal Eating-Drinking Syndromes”):ti,ab,kw OR (“Syndrome, Nocturnal Eating-Drinking”):ti,ab,kw OR (“Syndromes, Nocturnal Eating-Drinking”):ti,ab,kw OR (“Adjustment Sleep Disorder”):ti,ab,kw OR (“Adjustment Sleep Disorders”):ti,ab,kw OR (“Sleep Disorders, Adjustment”):ti,ab,kw OR (“Sleep Disorder, Adjustment”):ti,ab,kw OR (“Environmental Sleep Disorder”):ti,ab,kw OR (“Environmental Sleep Disorders”):ti,ab,kw OR (“Sleep Disorders, Environmental”):ti,ab,kw OR (“Sleep Disorder, Environmental”):ti,ab,kw) OR (MeSH descriptor: [Body Weight] explode all trees OR MeSH descriptor: [Weight Loss] explode all trees OR MeSH descriptor: [Weight Gain] explode all trees OR MeSH descriptor: [Body Weight Changes] explode all trees OR MeSH descriptor: [Body Mass Index] explode all tres OR MeSH descriptor: [Obesity] explode all trees OR MeSH descriptor: [Overweight] explode all trees OR ("body mass index"):ti,ab,kw OR ("body fatness"):ti,ab,kw OR ("weight

	change"):ti,ab,kw OR ("weight variability"):ti,ab,kw OR ("weight gain"):ti,ab,kw OR ("weight loss"):ti,ab,kw OR ("obesity"):ti,ab,kw OR ("overweight"):ti,ab,kw OR ("body weight"):ti,ab,kw OR ("adiposity"):ti,ab,kw OR ("fat mass"):ti,ab,kw OR ("body fat"):ti,ab,kw OR ("body size"):ti,ab,kw OR ("body composition"):ti,ab,kw OR ("central obesity"):ti,ab,kw)) AND ((“Chronotype”):ti,ab,kw OR (“Matutine Chronotype”):ti,ab,kw OR (“intermediate Chronotype”):ti,ab,kw OR ("Chronotherapies"):ti,ab,kw OR (“Circadian Rhythm Signaling Peptides and Proteins”):ti,ab,kw OR (“Circadian Clock Proteins and Peptides”):ti,ab,kw OR (“Circadian Rhythm Signaling Peptides”):ti,ab,kw OR (“Circadian Rhythm Signaling Proteins”):ti,ab,kw OR (“Circadian Clocks”):ti,ab,kw OR (“Circadian Clock”):ti,ab,kw OR (“Clock, Circadian”):ti,ab,kw OR (“Clocks, Circadian”):ti,ab,kw OR (“Circadian Clock System”):ti,ab,kw OR (“Circadian Clock Systems”):ti,ab,kw OR (“Clock System, Circadian”):ti,ab,kw OR (“Clock Systems, Circadian”):ti,ab,kw OR (“System, Circadian Clock”):ti,ab,kw OR (“Systems, Circadian Clock”):ti,ab,kw OR (“Circadian Timing System”):ti,ab,kw OR (“Circadian Timing Systems”):ti,ab,kw OR c(“System, Circadian Timing”):ti,ab,kw OR (“Systems, Circadian Timing”):ti,ab,kw OR (“Timing System, Circadian”):ti,ab,kw OR (“Timing Systems, Circadian”):ti,ab,kw OR (“Circadian Rhythms”):ti,ab,kw OR (“Rhythm, Circadian”):ti,ab,kw OR (“Rhythms, Circadian”):ti,ab,kw OR (“Twenty-Four Hour Rhythm”):ti,ab,kw OR (“Rhythm, Twenty-Four Hour”):ti,ab,kw OR (“Rhythms, Twenty-Four Hour”):ti,ab,kw OR (“Twenty Four Hour Rhythm”):ti,ab,kw OR (“Twenty-Four Hour Rhythms”):ti,ab,kw OR (“Nyctohemeral Rhythm”):ti,ab,kw OR (“Nyctohemeral Rhythms”):ti,ab,kw OR (“Rhythm, Nyctohemeral”):ti,ab,kw OR (“Rhythms, Nyctohemeral”):ti,ab,kw OR (“Nycthemeral Rhythm”):ti,ab,kw OR (“Nycthemeral Rhythms”):ti,ab,kwOR(“Rhythm,Nycthemeral”):ti,ab,kw OR (“Rhythms, Nycthemeral”):ti,ab,kw OR (“Diurnal Rhythm”):ti,ab,kw OR (“Diurnal Rhythms”):ti,ab,kw OR (“Rhythm, Diurnal”):ti,ab,kw OR(“Rhythms, Diurnal”):ti,ab,kw))
EBSCO	AB (((“Feeding and Eating Disorders” OR “Eating and Feeding Disorders” OR “Feeding Disorders” OR “Disorder, Feeding” OR “Disorders, Feeding” OR “Feeding Disorder” OR “Eating Disorders” OR “Disorder, Eating” OR “Disorders, Eating” OR “Eating Disorder” OR “Appetite Disorders” OR “Appetite Disorder” OR “Binge-Eating Disorder” OR “Binge Eating Disorder” OR “Binge-Eating Disorders” OR “Disorder, Binge-Eating” OR “Disorders, Binge-Eating” OR “Night Eating Syndrome” OR “Eating Syndrome, Night” OR “Eating Syndromes, Night” OR “Night Eating Syndromes” OR “Bulimia” OR “Bulimias” OR “Binge Eating” OR “Eating, Binge” OR “Food Addiction” OR “Food Addictions” OR “Compulsive Eating” OR “Eating, Compulsive” OR “Diet” OR “Diets” OR “Digestive System” OR “Alimentary System” OR “Dyssomnias” OR “Dyssomnia” OR “Sleep Disorders, Extrinsic” OR “Extrinsic Sleep Disorder” OR “Extrinsic Sleep Disorders” OR “Sleep Disorder, Extrinsic” OR “Limit-

	Setting Sleep Disorder” OR “Limit Setting Sleep Disorder” OR “Limit-Setting Sleep Disorders” OR “Sleep Disorders, Limit-Setting” OR “Sleep Disorder, Limit-Setting” OR “Sleep Disorder, Limit Setting” OR “Nocturnal Eating-Drinking Syndrome” OR “Eating-Drinking Syndrome, Nocturnal” OR “Eating-Drinking Syndromes, Nocturnal” OR “Nocturnal Eating Drinking Syndrome” OR “Nocturnal Eating-Drinking Syndromes” OR “Syndrome, Nocturnal Eating-Drinking” OR “Syndromes, Nocturnal Eating-Drinking” OR “Adjustment Sleep Disorder” OR “Adjustment Sleep Disorders” OR “Sleep Disorders, Adjustment” OR “Sleep Disorder, Adjustment” OR “Environmental Sleep Disorder” OR “Environmental Sleep Disorders” OR “Sleep Disorders, Environmental” OR “Sleep Disorder, Environmental”) OR (“Body Weight” OR “Weight Loss” OR “Weight Gain” OR “Body Weight Changes” OR “Body Mass Index” OR “Obesity” OR “Overweight” OR "body mass index" OR "body fatness" OR "weight change" OR "weght variability" OR "weight gain" OR "weight loss" OR "obesity" OR "overweight" OR "body weight" OR "adiposity" OR "fat mass" OR "body fat" OR "body size" OR "body composition" OR "central obesity")) AND (“Chronotype” OR “Matutine Chronotype” OR “intermediate Chronotype” OR "Chronotherapies" OR “Circadian Rhythm Signaling Peptides and Proteins” OR “Circadian Clock Proteins and Peptides” OR “Circadian Rhythm Signaling Peptides” OR “Circadian Rhythm Signaling Proteins” OR “Circadian Clocks” OR “Circadian Clock” OR “Clock, Circadian” OR “Clocks, Circadian” OR “Circadian Clock System” OR “Circadian Clock Systems” OR “Clock System, Circadian” OR “Clock Systems, Circadian” OR “System, Circadian Clock” OR “Systems, Circadian Clock” OR “Circadian Timing System” OR “Circadian Timing Systems” OR “System, Circadian Timing” OR “Systems, Circadian Timing” OR “Timing System, Circadian” OR “Timing Systems, Circadian” OR “Circadian Rhythms” OR “Rhythm, Circadian” OR “Rhythms, Circadian” OR “Twenty-Four Hour Rhythm” OR “Rhythm, Twenty-Four Hour” OR “Rhythms, Twenty-Four Hour” OR “Twenty Four Hour Rhythm” OR “Twenty-Four Hour Rhythms” OR “Nyctohemeral Rhythm” OR “Nyctohemeral Rhythms” OR “Rhythm, Nyctohemeral” OR “Rhythms, Nyctohemeral” OR “Nycthemeral Rhythm” OR “Nycthemeral Rhythms” OR “Rhythm, Nycthemeral” OR “Rhythms, Nycthemeral” OR “Diurnal Rhythm” OR “Diurnal Rhythms” OR “Rhythm, Diurnal” OR “Rhythms, Diurnal”))
EMBASE	((feeding:ab,ti AND 'eating disorders':ab,ti OR eating:ab,ti) AND 'feeding disorders':ab,ti OR 'feeding disorders':ab,ti OR 'disorder, feeding':ab,ti OR 'disorders, feeding':ab,ti OR 'feeding disorder':ab,ti OR 'eating disorders':ab,ti OR 'disorder, eating':ab,ti OR 'disorders, eating':ab,ti OR 'eating disorder':ab,ti OR 'appetite disorders':ab,ti OR 'appetite disorder':ab,ti OR 'binge-eating disorder':ab,ti OR 'binge eating disorder':ab,ti OR 'binge-eating disorders':ab,ti OR 'disorder, binge-eating':ab,ti OR 'disorders, binge-eating':ab,ti OR 'night eating syndrome':ab,ti OR 'eating syndrome, night':ab,ti OR 'eating syndromes, night':ab,ti OR 'night eating syndromes':ab,ti OR 'bulimia':ab,ti OR 'bulimias':ab,ti OR 'binge eating':ab,ti OR 'eating, binge':ab,ti OR

'food addiction':ab,ti OR 'food addictions':ab,ti OR 'compulsive eating':ab,ti OR 'eating, compulsive':ab,ti OR 'diet':ab,ti OR 'diets':ab,ti OR 'digestive system':ab,ti OR 'alimentary system':ab,ti OR 'dyssomnias':ab,ti OR 'dyssomnia':ab,ti OR 'sleep disorders, extrinsic':ab,ti OR 'extrinsic sleep disorder':ab,ti OR 'extrinsic sleep disorders':ab,ti OR 'sleep disorder, extrinsic':ab,ti OR 'limit-setting sleep disorder':ab,ti OR 'limit setting sleep disorder':ab,ti OR 'limit-setting sleep disorders':ab,ti OR 'sleep disorders, limit-setting':ab,ti OR 'sleep disorder, limit-setting':ab,ti OR 'sleep disorder, limit setting':ab,ti OR 'nocturnal eating-drinking syndrome':ab,ti OR 'eating-drinking syndrome, nocturnal':ab,ti OR 'eating-drinking syndromes, nocturnal':ab,ti OR 'nocturnal eating drinking syndrome':ab,ti OR 'nocturnal eating-drinking syndromes':ab,ti OR 'syndrome, nocturnal eating-drinking':ab,ti OR 'syndromes, nocturnal eating-drinking':ab,ti OR 'adjustment sleep disorder':ab,ti OR 'adjustment sleep disorders':ab,ti OR 'sleep disorders, adjustment':ab,ti OR 'sleep disorder, adjustment':ab,ti OR 'environmental sleep disorder':ab,ti OR 'environmental sleep disorders':ab,ti OR 'sleep disorders, environmental':ab,ti OR 'sleep disorder, environmental':ab,ti OR 'body weight changes':ab,ti OR 'body mass index':ab,ti OR 'body fatness':ab,ti OR 'weight change':ab,ti OR 'weght variability':ab,ti OR 'weight gain':ab,ti OR 'weight loss':ab,ti OR 'obesity':ab,ti OR 'overweight':ab,ti OR 'body weight':ab,ti OR 'adiposity':ab,ti OR 'fat mass':ab,ti OR 'body fat':ab,ti OR 'body size':ab,ti OR 'body composition':ab,ti OR 'central obesity':ab,ti) AND ((('chronotype':ab,ti OR 'matutine chronotype':ab,ti OR 'intermediate chronotype':ab,ti OR 'chronotherapies':ab,ti OR 'circadian rhythm signaling peptides':ab,ti) AND proteins:ab,ti OR 'circadian clock proteins':ab,ti) AND peptides:ab,ti OR 'circadian rhythm signaling peptides':ab,ti OR 'circadian rhythm signaling proteins':ab,ti OR 'circadian clocks':ab,ti OR 'circadian clock':ab,ti OR 'clock, circadian':ab,ti OR 'clocks, circadian':ab,ti OR 'circadian clock system':ab,ti OR 'circadian clock systems':ab,ti OR 'clock system, circadian':ab,ti OR 'clock systems, circadian':ab,ti OR 'system, circadian clock':ab,ti OR 'systems, circadian clock':ab,ti OR 'circadian timing system':ab,ti OR 'circadian timing systems':ab,ti OR 'system, circadian timing':ab,ti OR 'systems, circadian timing':ab,ti OR 'timing system, circadian':ab,ti OR 'timing systems, circadian':ab,ti OR 'circadian rhythms':ab,ti OR 'rhythm, circadian':ab,ti OR 'rhythms, circadian':ab,ti OR 'twenty-four hour rhythm':ab,ti OR 'rhythm, twenty-four hour':ab,ti OR 'rhythms, twenty-four hour':ab,ti OR 'twenty four hour rhythm':ab,ti OR 'twenty-four hour rhythms':ab,ti OR 'nyctohemeral rhythm':ab,ti OR 'nyctohemeral rhythms':ab,ti OR 'rhythm, nyctohemeral':ab,ti OR 'rhythms, nyctohemeral':ab,ti OR 'nycthemeral rhythm':ab,ti OR 'nycthemeral rhythms':ab,ti OR 'rhythm, nycthemeral':ab,ti OR 'rhythms, nycthemeral':ab,ti OR 'diurnal rhythm':ab,ti OR 'diurnal rhythms':ab,ti OR 'rhythm, diurnal':ab,ti OR 'rhythms, diurnal':ab,ti)

Pubmed (((“Feeding and Eating Disorders” OR “Eating and Feeding Disorders” OR “Feeding Disorders” OR “Disorder, Feeding” OR “Disorders, Feeding” OR “Feeding Disorder” OR “Eating Disorders” OR “Disorder, Eating” OR “Disorders, Eating” OR “Eating

Disorder" OR "Appetite Disorders" OR "Appetite Disorder" OR "Binge-Eating Disorder" OR "Binge Eating Disorder" OR "Binge-Eating Disorders" OR "Disorder, Binge-Eating" OR "Disorders, Binge-Eating" OR "Night Eating Syndrome" OR "Eating Syndrome, Night" OR "Eating Syndromes, Night" OR "Night Eating Syndromes" OR "Bulimia" OR "Bulimias" OR "Binge Eating" OR "Eating, Binge" OR "Food Addiction" OR "Food Addictions" OR "Compulsive Eating" OR "Eating, Compulsive" OR "Diet" OR "Diets" OR "Digestive System" OR "Alimentary System" OR "Dyssomnias" OR "Dyssomnia" OR "Sleep Disorders, Extrinsic" OR "Extrinsic Sleep Disorder" OR "Extrinsic Sleep Disorders" OR "Sleep Disorder, Extrinsic" OR "Limit-Setting Sleep Disorder" OR "Limit Setting Sleep Disorder" OR "Limit-Setting Sleep Disorders" OR "Sleep Disorders, Limit-Setting" OR "Sleep Disorder, Limit-Setting" OR "Sleep Disorder, Limit Setting" OR "Nocturnal Eating-Drinking Syndrome" OR "Eating-Drinking Syndrome, Nocturnal" OR "Eating-Drinking Syndromes, Nocturnal" OR "Nocturnal Eating Drinking Syndrome" OR "Nocturnal Eating-Drinking Syndromes" OR "Syndrome, Nocturnal Eating-Drinking" OR "Syndromes, Nocturnal Eating-Drinking" OR "Adjustment Sleep Disorder" OR "Adjustment Sleep Disorders" OR "Sleep Disorders, Adjustment" OR "Sleep Disorder, Adjustment" OR "Environmental Sleep Disorder" OR "Environmental Sleep Disorders" OR "Sleep Disorders, Environmental" OR "Sleep Disorder, Environmental") OR ("Body Weight" OR "Weight Loss" OR "Weight Gain" OR "Body Weight Changes" OR "Body Mass Index" OR "Obesity" OR "Overweight" OR "body mass index" OR "body fatness" OR "weight change" OR "weght variability" OR "weight gain" OR "weight loss" OR "obesity" OR "overweight" OR "body weight" OR "adiposity" OR "fat mass" OR "body fat" OR "body size" OR "body composition" OR "central obesity")) AND ("Chronotype" OR "Matutine Chronotype" OR "intermediate Chronotype" OR "Chronotherapies" OR "Circadian Rhythm Signaling Peptides and Proteins" OR "Circadian Clock Proteins and Peptides" OR "Circadian Rhythm Signaling Peptides" OR "Circadian Rhythm Signaling Proteins" OR "Circadian Clocks" OR "Circadian Clock" OR "Clock, Circadian" OR "Clocks, Circadian" OR "Circadian Clock System" OR "Circadian Clock Systems" OR "Clock System, Circadian" OR "Clock Systems, Circadian" OR "System, Circadian Clock" OR "Systems, Circadian Clock" OR "Circadian Timing System" OR "Circadian Timing Systems" OR "System, Circadian Timing" OR "Systems, Circadian Timing" OR "Timing System, Circadian" OR "Timing Systems, Circadian" OR "Circadian Rhythms" OR "Rhythm, Circadian" OR "Rhythms, Circadian" OR "Twenty-Four Hour Rhythm" OR "Rhythm, Twenty-Four Hour" OR "Rhythms, Twenty-Four Hour" OR "Twenty Four Hour Rhythm" OR "Twenty-Four Hour Rhythms" OR "Nyctohemeral Rhythm" OR "Nyctohemeral Rhythms" OR "Rhythm, Nyctohemeral" OR "Rhythms, Nyctohemeral" OR "Nycthemeral Rhythm" OR "Nycthemeral Rhythms" OR "Rhythm, Nycthemeral" OR "Rhythms, Nycthemeral" OR "Diurnal Rhythm" OR "Diurnal Rhythms" OR "Rhythm, Diurnal" OR "Rhythms, Diurnal"))

Web of Science	((TS=((“Feeding and Eating Disorders” OR “Eating and Feeding Disorders” OR “Feeding Disorders” OR “Disorder, Feeding” OR “Disorders, Feeding” OR “Feeding Disorder” OR “Eating Disorders” OR “Disorder, Eating” OR “Disorders, Eating” OR “Eating Disorder” OR “Appetite Disorders” OR “Appetite Disorder” OR “Binge-Eating Disorder” OR “Binge Eating Disorder” OR “Binge-Eating Disorders” OR “Disorder, Binge-Eating” OR “Disorders, Binge-Eating” OR “Night Eating Syndrome” OR “Eating Syndrome, Night” OR “Eating Syndromes, Night” OR “Night Eating Syndromes” OR “Bulimia” OR “Bulimias” OR “Binge Eating” OR “Eating, Binge” OR “Food Addiction” OR “Food Addictions” OR “Compulsive Eating” OR “Eating, Compulsive” OR “Diet” OR “Diets” OR “Digestive System” OR “Alimentary System” OR “Dyssomnias” OR “Dyssomnia” OR “Sleep Disorders, Extrinsic” OR “Extrinsic Sleep Disorder” OR “Extrinsic Sleep Disorders” OR “Sleep Disorder, Extrinsic” OR “Limit-Setting Sleep Disorder” OR “Limit Setting Sleep Disorder” OR “Limit-Setting Sleep Disorders” OR “Sleep Disorders, Limit-Setting” OR “Sleep Disorder, Limit-Setting” OR “Sleep Disorder, Limit Setting” OR “Nocturnal Eating-Drinking Syndrome” OR “Eating-Drinking Syndrome, Nocturnal” OR “Eating-Drinking Syndromes, Nocturnal” OR “Nocturnal Eating Drinking Syndrome” OR “Nocturnal Eating-Drinking Syndromes” OR “Syndrome, Nocturnal Eating-Drinking” OR “Syndromes, Nocturnal Eating-Drinking” OR “Adjustment Sleep Disorder” OR “Adjustment Sleep Disorders” OR “Sleep Disorders, Adjustment” OR “Sleep Disorder, Adjustment” OR “Environmental Sleep Disorder” OR “Environmental Sleep Disorders” OR “Sleep Disorders, Environmental” OR “Sleep Disorder, Environmental”))) OR TS=((“Body Weights” OR “Weight, Body” OR “Weights, Body” OR “Loss, Weight” OR “Losses, Weight” OR “Weight Losses” OR “Weight Reduction” OR “Reduction, Weight” OR “Reductions, Weight” OR “Weight Reductions” OR “Gain, Weight” OR “Gains, Weight” OR “Weight Gains” OR “Body Weight Change” OR “Change, Body Weight” OR “Changes, Body Weight” OR “Weight Change, Body” OR “Weight Changes, Body” OR “Index, Body Mass” OR “Quetelet Index” OR “Index, Quetelet” OR “Quetelet's Index” OR “Quetelets Index” OR “Obesity” OR “Overweight” OR "body mass index" OR "body fatness" OR "weight change" OR "weight variability" OR "weight gain" OR "weight loss" OR "obesity" OR "overweight" OR "body weight" OR "adiposity" OR "fat mass" OR "body fat" OR "body size" OR "body composition" OR "central obesity"))) AND TS=((“Chronotype” OR “Matutine Chronotype” OR “intermediate Chronotype” OR "Chronotherapies" OR “Circadian Rhythm Signaling Peptides and Proteins” OR “Circadian Clock Proteins and Peptides” OR “Circadian Rhythm Signaling Peptides” OR “Circadian Rhythm Signaling Proteins” OR “Circadian Clocks” OR “Circadian Clock” OR “Clock, Circadian” OR “Clocks, Circadian” OR “Circadian Clock System” OR “Circadian Clock Systems” OR “Clock System, Circadian” OR “Clock Systems, Circadian” OR “System, Circadian Clock” OR “Systems, Circadian Clock” OR “Circadian Timing System” OR “Circadian Timing Systems” OR “System, Circadian

Timing" OR "Systems, Circadian Timing" OR "Timing System, Circadian" OR "Timing Systems, Circadian" OR "Circadian Rhythms" OR "Rhythm, Circadian" OR "Rhythms, Circadian" OR "Twenty-Four Hour Rhythm" OR "Rhythm, Twenty-Four Hour" OR "Rhythms, Twenty-Four Hour" OR "Twenty Four Hour Rhythm" OR "Twenty-Four Hour Rhythms" OR "Nyctohemeral Rhythm" OR "Nyctohemeral Rhythms" OR "Rhythm, Nyctohemeral" OR "Rhythms, Nyctohemeral" OR "Nycthemeral Rhythm" OR "Nycthemeral Rhythms" OR "Rhythm, Nycthemeral" OR "Rhythms, Nycthemeral" OR "Diurnal Rhythm" OR "Diurnal Rhythms" OR "Rhythm, Diurnal" OR "Rhythms, Diurnal"))

Table S2. Quality Assessment for Observational Cross-sectional Studies (National Heart, Lung, and Blood Institute, 2021)

Criteria	19	20	21	22	23	24	25	26
1. Was the research question or objective in this paper clearly stated?	**Yes**	**Yes**	**Yes**	**Yes**	**Yes**	**Yes**	**Yes**	**Yes**
2. Was the study population clearly specified and defined?	**Yes**	**Yes**	**Yes**	**Yes**	**Yes**	**Yes**	**Yes**	**Yes**
3. Was the participation rate of eligible persons at least 50%?	**Yes**	**Yes**	**Yes**	**Yes**	**Yes**	**Yes**	**Yes**	**Yes**
4. Were all the subjects selected or recruited from the same or similar populations (including the same time period)?	**Yes**	**Yes**	**Yes**	**Yes**	**Yes**	**Yes**	**Yes**	**Yes**
Were inclusion and exclusion criteria for being in the study prespecified and applied uniformly to all participants?	**Yes**	**Yes**	Yes	**Yes**	**Yes**	**Yes**	**No**	**Yes**
5. Was a sample size justification, power description, or variance and effect estimates provided?	**No**	**No**	**No**	**No**	**No**	**No**	**No**	**No**
6. For the analyses in this paper, were the exposure(s) of interest measured prior to the outcome(s) being measured?	**No**	**No**	**No**	**No**	**No**	**No**	**No**	**No**
7. Was the timeframe sufficient so that one could reasonably expect to see an association between exposure and outcome if it existed?	**No**	**No**	**No**	**No**	**No**	**No**	**No**	**No**
8. For exposures that can vary in amount or level, did the study examine different levels of the exposure as related to the outcome (e.g., categories of exposure, or exposure measured as continuous variable)?	**Yes**	**Yes**	**Yes**	**Yes**	**Yes**	**Yes**	**Yes**	**Yes**
9. Were the exposure measures (independent variables) clearly defined, valid, reliable, and implemented consistently across all study participants?	**Yes**	**Yes**	**Yes**	**Yes**	**Yes**	**Yes**	**Yes**	**Yes**
10. Was the exposure(s) assessed more than once over time?	**NA**	**NA**	**NA**	**NA**	**NA**	**NA**	**NA**	**NA**
11. Were the outcome measures (dependent variables) clearly defined, valid, reliable, and implemented consistently across all study participants?	**Yes**	**Yes**	**Yes**	**Yes**	**Yes**	**Yes**	**Yes**	**Yes**
12. Were the outcome assessors blinded to the exposure status of participants?	**NA**	**NA**	**NA**	**NA**	**NA**	**NA**	**NA**	**NA**
13. Was loss to follow-up after baseline 20% or less?	**Yes**	**Yes**	**Yes**	**Yes**	**Yes**	**Yes**	**Yes**	**Yes**
14. Were key potential confounding variables measured and adjusted statistically for their impact on the relationship between exposure(s) and outcome(s)?	**No**	**Yes**	**No**	**Yes**	**No**	**Yes**	**Yes**	**Yes**
Quality Rating (Good, Fair, or Poor)	**Fair**	**Good**	**Fair**	**Good**	**Fair**	**Good**	**Fair**	**Good**

*CD, cannot determine; NA, not applicable; NR, not reported

Table S3. Quality Assessment for Observational Cross-sectional Studies (National Heart, Lung, and Blood Institute, 2021)

Criteria	29	10	27	12	28
1. Was the research question or objective in this paper clearly stated?	**Yes**	**Yes**	**Yes**	**Yes**	**Yes**
2. Was the study population clearly specified and defined?	**Yes**	**Yes**	**Yes**	**Yes**	**Yes**
3. Was the participation rate of eligible persons at least 50%?	**Yes**	**Yes**	**Yes**	**Yes**	**Yes**
4. Were all the subjects selected or recruited from the same or similar populations (including the same time period)?	**Yes**	**Yes**	**Yes**	**Yes**	**Yes**
Were inclusion and exclusion criteria for being in the study prespecified and applied uniformly to all participants?	**Yes**	**Yes**	**Yes**	**Yes**	**Yes**
5. Was a sample size justification, power description, or variance and effect estimates provided?	**Yes**	**No**	**No**	**No**	**No**
6. For the analyses in this paper, were the exposure(s) of interest measured prior to the outcome(s) being measured?	**NA**	**No**	**No**	**No**	**No**
7. Was the timeframe sufficient so that one could reasonably expect to see an association between exposure and outcome if it existed?	**NA**	**No**	**No**	**No**	**No**
8. For exposures that can vary in amount or level, did the study examine different levels of the exposure as related to the outcome (e.g., categories of exposure, or exposure measured as continuous variable)?	**Yes**	**Yes**	**Yes**	**Yes**	**Yes**
9. Were the exposure measures (independent variables) clearly defined, valid, reliable, and implemented consistently across all study participants?	**Yes**	**Yes**	**Yes**	**Yes**	**Yes**
10. Was the exposure(s) assessed more than once over time?	**NA**	**NA**	**NA**	**NA**	**NA**
11. Were the outcome measures (dependent variables) clearly defined, valid, reliable, and implemented consistently across all study participants?	**Yes**	**Yes**	**Yes**	**Yes**	**Yes**
12. Were the outcome assessors blinded to the exposure status of participants?	**NA**	**NA**	**NA**	**NA**	**NA**
13. Was loss to follow-up after baseline 20% or less?	**Yes**	**Yes**	**Yes**	**Yes**	**Yes**
14. Were key potential confounding variables measured and adjusted statistically for their impact on the relationship between exposure(s) and outcome(s)?	**Yes**	**Yes**	**No**	**No**	**No**
Quality Rating (Good, Fair, or Poor)	**Good**	**Good**	**Fair**	**Fair**	**Fair**

*CD, cannot determine; NA, not applicable; NR, not reported

Table S4. Quality Assessment of Case-Control Studies (National Heart, Lung, and Blood Institute, 2021)

Criteria	30	31
1. Was the research question or objective in this paper clearly stated and appropriate?	**Yes**	**Yes**
2. Was the study population clearly specified and defined?	**Yes**	**Yes**
3. Did the authors include a sample size justification?	**No**	**No**
4. Were controls selected or recruited from the same or similar population that gave rise to the cases (including the same timeframe)?	**Yes**	**Yes**
5. Were the definitions, inclusion and exclusion criteria, algorithms or processes used to identify or select cases and controls valid, reliable, and implemented consistently across all study participants?	**Yes**	**Yes**
6. Were the cases clearly defined and differentiated from controls?	**Yes**	**Yes**
7. If less than 100 percent of eligible cases and/or controls were selected for the study, were the cases and/or controls randomly selected from those eligible?	**NA**	**NA**
8. Was there use of concurrent controls?	**NR**	**NR**
9. Were the investigators able to confirm that the exposure/risk occurred prior to the development of the condition or event that defined a participant as a case?	**NR**	**NR**
10. Were the measures of exposure/risk clearly defined, valid, reliable, and implemented consistently (including the same time period) across all study participants?	**NR**	**NR**
11. Were the assessors of exposure/risk blinded to the case or control status of participants?	**NR**	**NR**
12. Were key potential confounding variables measured and adjusted statistically in the analyses? If matching was used, did the investigators account for matching during study analysis?	**Yes**	**Yes**
Quality Rating (Good, Fair, or Poor)	**Fair**	**Fair**

*CD, cannot determine; NA, not applicable; NR, not reported
